# Unidirectional propagation of the Bloch surface wave excited by the spinning magnetic dipole in two-dimensional photonic crystal slab

**DOI:** 10.1038/s41598-021-98056-y

**Published:** 2021-09-16

**Authors:** Li-Ming Zhao, Yun-Song Zhou

**Affiliations:** Department of Physics, Capital Normal University, Beijing, 100048 China

**Keywords:** Photonic crystals, Integrated optics

## Abstract

The photonic spin Hall effect (PSHE) can be realized in a photonic crystal (PC) slab, that is, the unidirectional Bloch surface wave can propagate along the surface of the PC slab under the excitation of elliptical polarized magnetic dipole. It is further proved that PSHE is caused by the interference of the component surface waves excited by the different components of the incident light, which is the so called component wave interference (CWI) theory. In addition, we also find that the spin of the surface wave oscillates periodically in space, and the oscillation period is a unit cell. In a unit cell, the average spin keeps the spin orbit locked. The results show that the spin separation can also be modulated by the position and the polarization state of the magnetic dipole.

Photonic spin Hall effect (PSHE) can separate photons according to different spin characteristics, which provides a new idea for the design of photonic devices and has important application prospects^1–8^. In 2013, Zayats et al. observed a new kind of PSHE in metal system, and found that surface plasmon polariton (SPP) can propagate unidirectionally, and the propagation direction can be controlled by the incidence of circularly polarized light^9^. Different from the traditional PSHE, the new PSHE has a strong Hall effect, which obviously separates the beams with different spin. The main explanations about PSHE are wavevector matching (WVM)^10,11^ and spin coupling theories^12^. For SPP, its spin is a transverse angular momentum, which has a chirality relationship with the surface and transmission direction. This chirality is an invariant, commonly known as spin orbit locking^13–19^. According to the spin coupling theory, the incident spin determines the spin of SPP, and then the spin of SPP determines the propagation direction of SPP. Recently, it has been found that the asymmetric scattering of PSHE is caused the component wave interference (CWI) of electric or magnetic dipoles^20–22^. According to CWI theory, the propagation direction of SPP is determined by the incident spin, and then the spin of SPP is determined by the spin orbit locking of SPP.

The surface waves in photonic crystal (PC) are the Bloch surface waves composed of zero order and higher order surface modes. In recent years, PSHE can be observed in two-dimensional (2D) PC^23^, and an open cavity was regarded as a scatterer. As we all know, an electric dipolar source or magnetic dipolar source can be realized experimentally by illuminating any small scatterer^10,24^, therefore, the scattering of scatterer is equivalent to the radiation of the dipole source.

Based on the above works, we use a magnetic dipole source instead of the scatterer as a general theoretical model to study theoretically the following two issues: Whether the mechanism of generating PSHE by Bloch surface waves is similar to that of SPP? Can the spin orbit locking always be satisfied? It is found that the asymmetric scattering of PSHE of the Bloch surface wave results from the CWI theory, which is affected by the polarization and the position of the magnetic dipole. It is further found that there exists the locked relationship between average spin with orbit.

## Radiation of magnetic dipole

**Figure 1:**
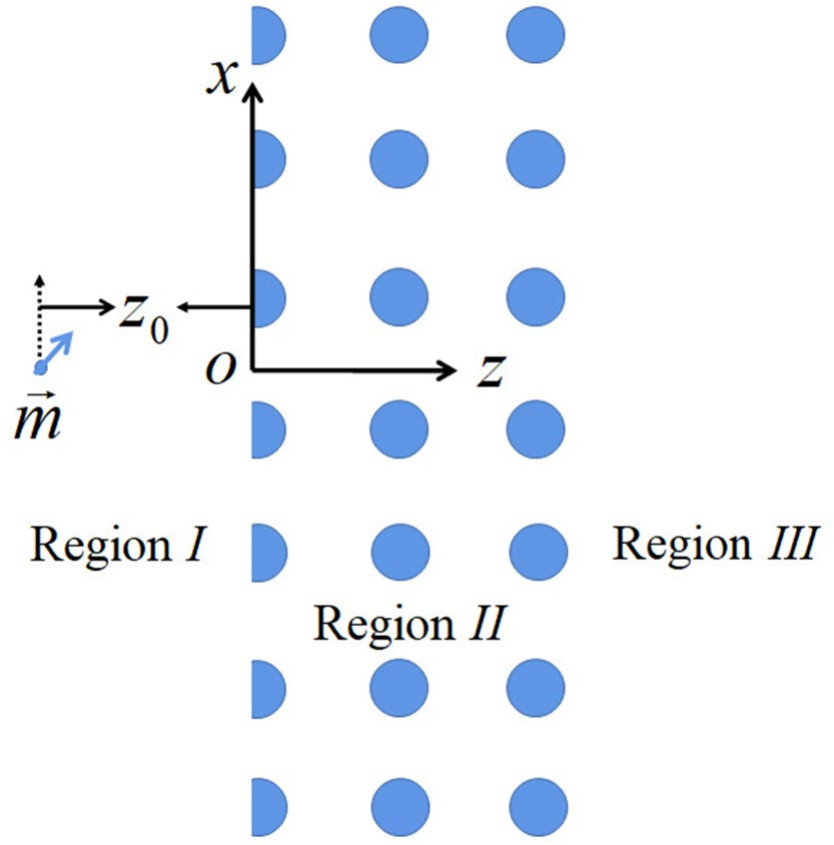
Sketch map of 2D PC slab, a magnetic dipole pillar $\vec{m}$ is located at $x=0$ and $z=z_0$.

The system investigated is a PC slab, it can divide the space into three regions, as shown in Fig. 1. The PC slab is located in region *II* and surrounded by air on either side (referred to regions I and III). The PC consists of a square lattice (with lattice constant a) of cylinders (with radius *r*). The periodic plane layers are laid on the xz plane and the cylinders are infinite along the y-direction, and the periodic layers along z-direction are finite, while along x-direction are infinite. In order to generate the interface states, the rods in the leftmost layer have been truncated along their diameter into a semicircular shape. In this paper, we just consider the E-polarized interface states, which can be excited by a magnetic dipole $\vec{m}$. Now, it is assumed that there is a magnetic dipole cylinder located in region *I* at $x=0$ and $z=z_0$ , and the interface between Region *I* and *II* is set as $z=0$. The magnetic dipole can radiate the electromagnetic wave, and the corresponding magnetic vector potential can be written as

1 $$\begin{aligned} \vec{A}(x,z,\vec{m})=\frac{i \mu _{0}}{4 \pi } \int ^{\infty }_{-\infty } dy (\vec{k}\times \vec{m})\frac{e^{ik|\vec{\rho}-y\hat{y}|}}{|\vec{\rho}-y\hat{y}|}=\frac{- \mu _{0} }{4 \pi }\int ^{\infty }_{-\infty } (\vec{k}\times \vec{m})\frac{dk_x}{k_z}e^{i(k_x x+k_z |z-z_0|)}, \end{aligned}$$

here, $\vec{\rho}=x\hat{x}+(z-z_0)\hat{z}$, $\vec{m}=(m_x, 0, m_z)$, and $k_z=\sqrt{k^2-k^2_x}$, where *k* is the wavenumber of the radiation field. According to Maxwell equation

$$\vec{H}=\frac{1}{ \mu _{0}}\nabla \times \vec{A}=\frac{-1}{4 \pi }\int ^{\infty }_{-\infty } \frac{dk_x}{k_z}i\vec{k}\times (\vec{k}\times \vec{m})e^{i(k_x x+k_z (z-z_0))}.$$

Here, we just consider the region $z>z_0$, and considering $\vec{k}=(k_x, 0, k_z)$ and $\vec{k}\times (\vec{k}\times \vec{m})=(k_x k_z m_z-k_{z}^{2} m_x)\hat{x}+(k_x k_z m_x-k_{x}^{2} m_z)\hat{z}$, $\vec{H}$ can be rewritten as

$$\vec{H}=\frac{-i }{4 \pi }\int ^{\infty }_{-\infty } \frac{dk_x}{k_z}[(k_x k_z m_z-k_{z}^{2} m_x)\hat{x}+(k_x k_z m_x-k_{x}^{2} m_z)\hat{z}]e^{i(k_x x+k_z (z-z_0))},$$

and the corresponding electric field is

$$\begin{aligned} \vec{E}=\frac{i}{ \omega \epsilon _0 } \nabla \times \vec{H}=\frac{ik^{2}}{4 \pi \omega \epsilon _0 }\int ^{\infty }_{-\infty } \frac{dk_x}{k_z} (k_x m_z-k_z m_x)\hat{y}e^{i(k_x x+k_z (z-z_0))}, \end{aligned}$$

where $\varepsilon _{0}$ and $\mu _{0}$ are permittivity and permeability of the air.

When the electromagnetic wave propagates at the interface of the PC slab, the corresponding incident field can be rewritten as

2 $$\begin{aligned} \vec{E}=\frac{ik^{2}}{4 \pi \omega \epsilon _0 }\sum _{n}\int ^{\pi }_{-\pi } \frac{dk_x}{k_{nz}} [(k_x+nG_x) m_z-k_{nz} m_x)]e^{i[(k_x+nG_x) x+k_{nz} (z-z_0)]}\hat{y}. \end{aligned}$$

here, $k_x$ is the Bloch wave vector and it is in the first Brillouin Zone (FBZ), and correspondingly, the $n-th$ order incident coefficients is $A_1^{n}= \frac{1}{k_{nz}} [(k_x+nG_x) m_z-k_{nz} m_x)]e^{-ik_{nz}z_{0}}$. The corresponding reflection field can be written as

3 $$\begin{aligned} \vec{E}_r(x,z)= \frac{ik^{2}}{4 \pi \omega \epsilon _0 }\sum _{n}\int ^{\pi }_{-\pi } dk_x B_1^{n}e^{i[(k_x+nG_x) x-k_{nz} z]}\hat{y}, \end{aligned}$$

here $B_1^{n}$ can be obtained by Eq. (A6).

Now, we assume the surface wave $\vec{E}_{SW}=\vec{E}_r+\vec{E}_{pr}$, and $\vec{E}_{pr}$ is the radiation field and $\vec{E}_{pr}=\frac{ik^{2}}{4 \pi \omega \epsilon _0 }\sum _{n}\int ^{\pi }_{-\pi } dk_x A_1^{n} e^{i[(k_x+nG_x) x-k_{nz}z]}\hat{y}.$ Thus, the surface wave is

4 $$\begin{aligned} \vec{E}_{SW}= \frac{ik^{2}}{4 \pi \omega \epsilon _0 }\sum _{n}\int ^{\pi }_{-\pi } dk_x (A_1^{n}+B_1^{n})e^{i[(k_x+nG_x) x-k_{nz} z]}\hat{y}. \end{aligned}$$

Once $\vec{E}_{SW}$ is known, the power of the surface wave in Region *I* is

5 $$\begin{aligned} \bar{P}=\int _{-\infty }^{0} dz 0.5Re(\frac{1}{i\mu _{0}\omega }E^{*}_{SW}\frac{\partial E_{SW}}{\partial x}), \end{aligned}$$

and the corresponding spin angular momentum density of surface wave can be written as^25,26^

6 $$\begin{aligned} \vec{S}=\frac{1}{4\omega }Im(\vec{D}^{*}_{SW}\times \vec{E}_{SW}+\vec{B}^{*}_{SW}\times \vec{H}_{SW}). \end{aligned}$$

For E-polarized field, Eq. (6) can be rewritten as

7 $$\begin{aligned} \vec{S}=\frac{1}{4\omega }Im(\vec{B}^{*}_{SW}\times \vec{H}_{SW}), \end{aligned}$$

and the corresponding energy density of the surface wave is

8 $$\begin{aligned} W=\frac{1}{4} \mu _{0} |H_{SW}|^{2}. \end{aligned}$$

According to Eqs. (7) and (8), we can obtain the average spin for one photon is

9 $$\begin{aligned} \bar{s}=\frac{Im(\vec{H}^{*}\times \bar{H})}{|H|^{2}}\hbar . \end{aligned}$$

## Results and analysis

**Figure 2:**
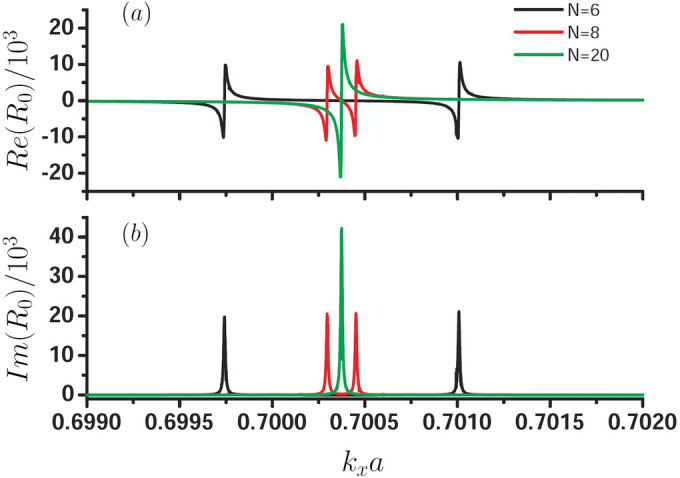
Reflection coefficients $R_0$ for $\omega =0.3 (2\pi c/a)$ as a function of $k_x$ for different periodic layer number *N* of the PC slab, black curve for $N=6$, red curve for $N=8$, and green curve for $N=20$, (**a**) is the real part of $R_0$, and (**b**) is the imaginary part of $R_0.$

We assume that the two-dimensional PC is composed of a square lattice of dielectric cylinder with a dielectric constant 11.56 and radius $$ r = 0.2 a$$. We have calculated the band structure by using the plan-wave expansion method and found that the photonic band gap (PBG) of E-polarized field is located in the circular frequency range of $$0.286-0.42 (2\pi c/a)$$. In Fig. 2, we first investigate the reflection coefficients $$R_0$$ (It can be obtained by Eq. (A9)) as a function of $$k_x$$ for different period layer number *N* of the finite PC slab when $$\omega =0.3 (2\pi c/a)$$, black curve for $$N=6$$, red curve for $$N=8$$, and green curve for $$N=20$$. Due to $$R_{0}$$ is a complex number, the variation of real part of $$R_0$$ denoted by $$Re(R_0)$$ with $$k_x$$ is presented in Fig. 2a and the variation of imaginary part of $$R_0$$ denoted by $$Im(R_0)$$ with $$k_x$$ is shown in Fig. 2b. It is noting that the reflection wave is the superposition of *n* plan waves, the corresponding wave vectors along *x* direction are $$k_x+m G_x$$, here $$m=-n/2,-n/2+1,...0,1,...n/2$$. Therefore, there are *n* corresponding reflection coefficients. The calculation results show that the $$0-th$$ reflection coefficient is the largest, for the higher-order reflection coefficients, their values are decreases rapidly. Moreover, the variation curves of other higher-order reflection coefficients with $$k_x$$ are similar with that of the $$0-th$$ reflection coefficient, so we only give the variation of $$0-th$$ reflection coefficient $$R_{0}$$ with $$k_x$$ in Fig. 2. It should be emphasized that the curve $$R_{0}$$ with $$k_x$$ is symmetrically distributed with respect to $$k_x =0$$. It is found that there are two singular points for the reflection coefficients in the case of $$N=6$$ and $$N=8$$, for $$N=6$$, the singular point are $$k_x a=0.69975$$ and 0.70101, and for $$N=8$$, the corresponding singular points are 0.7003 and 0.70045. We further found that with the increase of *N*, the distance of the two singular points are smaller, and when $$N=20$$, the singular point is about $$k_x a=0.70038$$ and the curve of the reflection coefficient is similar to that of SPP, and the singular point is corresponding to the interface modes. We also obtain the interface modes for other frequencies by the singular behavior of the reflection coefficient, and finally, we find that our results are similar with Fig. 4 in Ref.^27^. It is worth noting that the reflection coefficient can also be regarded as the excitation rate of the surface state, when its absolute value is larger, the ability to excite surface states is stronger.

**Figure 3:**
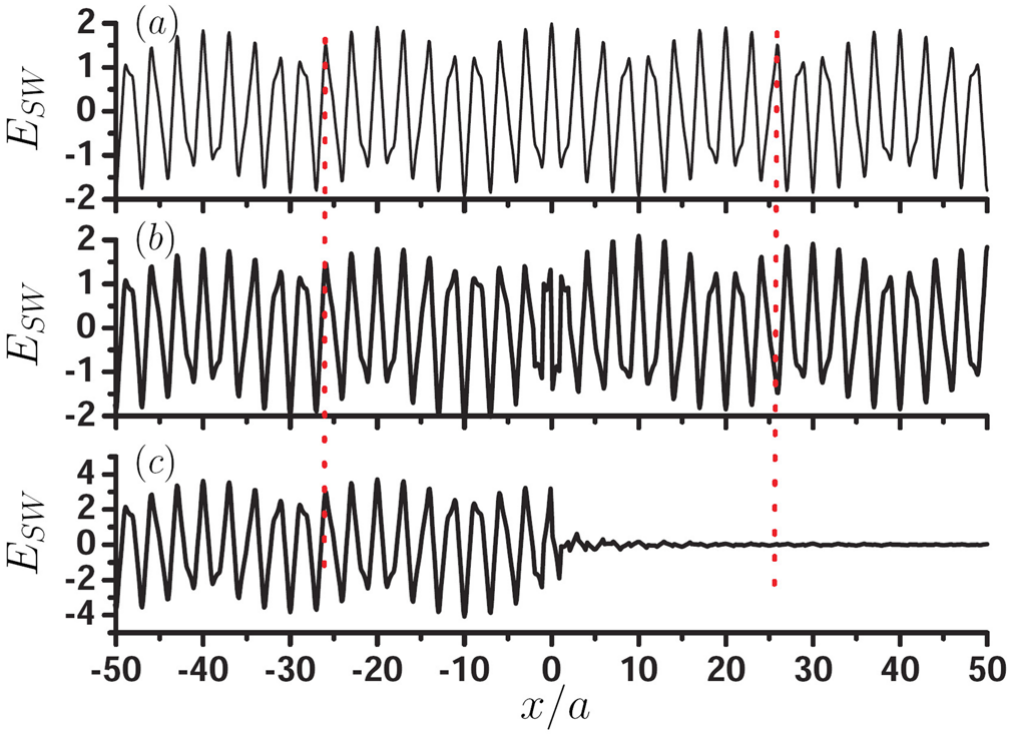
Surface field at $z=0$ as a function of *x* for different $\vec{m}$, (**a**) for $\vec{m}=cos58^{\circ }e^{-i\omega t} \hat{x}$, (**b**) for $\vec{m}=isin58^{\circ }e^{-i\omega t} \hat{z}$, and (**c**) for $\vec{m}=(cos58^{\circ } \hat{x}+isin58^{\circ } \hat{z})e^{-i\omega t}$.

Now, we investigate the asymmetric scattering (unidirectional scattering) of PSHE for the above PC slab in the case of $N=6$. For simplicity, let’s assume that a magnetic dipole $\vec{m}=(cos\theta \hat{x}+isin\theta \hat{z})e^{-i\omega t}$ illuminates the system, and $\theta$ is polarization angle, the position of $\vec{m}$ is set as $z_0=-0.1a$. Figure 3 shows the field at the interface (arbitrary unit (A. U)) as a function of x for different $\vec{m}$, (a) for $\vec{m}=cos58^{\circ }e^{-i\omega t} \hat{x}$, (b) for $\vec{m}=isin58^{\circ }e^{-i\omega t} \hat{z}$, and (c) for $\vec{m}=(cos58^{\circ } \hat{x}+isin58^{\circ } \hat{z})e^{-i\omega t}$, here $\omega =0.3 (2\pi c/a)$. As can be seen from Fig. 3a, the surface field excited by $m_x$ is the even function of $x=0$, and the surface field excited by $m_z$ is a odd function of $x=0$, as shown in Fig. 3b. For the curve in Fig. 3c, it comes from the superposition of the curves in Fig. 3a,b, obviously, this curve presents an asymmetric behaviors. There are two vertical red dashed lines through the three graphs in Fig. 3, the left one shows that the two component waves (Fig. 3a,b) in $x<0$ region are instructive interference, so the total wave propagating to the left is strong. Similarly, the right one are interference destructively, so the total wave (Fig. 3c) propagating to the right is suppressed. In other words, the asymmetric scattering results from the superposition of fields with different parity excited by two different components of magnetic dipoles, which is so-called CWI principle.

We can also see that the surface field in Fig. 3 shows some periodicity, and the period is about 20*a*. It can be seen from Eq. (4) that the surface field is the superposition of Bloch waves with different Bloch wave vectors $k_x$. The larger the reflection coefficient, the greater the contribution to the surface field. According to Fig. 1, the Bloch waves of $k_x a=\pm 0.7 \pi$ have the largest contribution on the surface field, and the superposition of the two component fields of $k_x x=\pm 0.7 \pi$ can get $cos{0.7 \pi a}$. In addition, due to the spatial periodicity of PCs, the period should be an integral multiple of the lattice constant. Therefore, we can finally obtain the corresponding period of the surface field is $\frac{7\times 2\pi }{0.7 \pi }a=20a$. We also calculate the surface field for $\omega =0.315 (2\pi c/a)$, when $k_x a=\pm 0.8\pi$ , the reflection coefficient is the largest, and we finally find that the period of the corresponding surface field is $\frac{2\times 2\pi }{0.8\pi }a=5a$.

**Figure 4:**
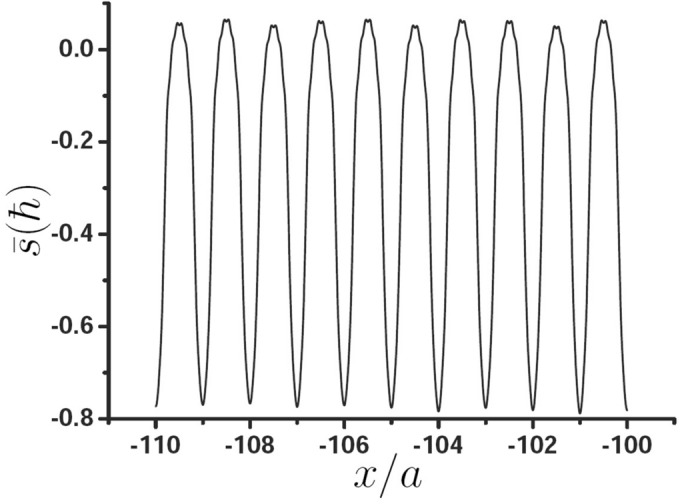
Variation of average spin $\bar{s}$ with *x*.

Now, we study the spin properties of the surface wave in the region of $x<0$, and the parameters are the same as those in Fig. 3c. The average spin of one photon of the surface wave can be obtained by Eq. (9). Figure 4 shows the variation of average spin $\bar{s}$ with *x*, it can be seen that the spin oscillates with a period of lattice constant *a*, which is different from the spin of the SPP, it is a constant and independent of position. It can be observed that in most regions (from -0.34a to 0.34a in a cell), the spin is less than 0 and has a large absolute value. Obviously, when $x<0$, the average spin in the periodic unit is always less than 0, which means that the average spin and orbit are locked.

Now, we define the spin separation, it can be defined as $\eta =\frac{\bar{P}_{+}}{\bar{P}_{+}+|\bar{P}_{-}|}$, here, $\bar{P}$ is the average power in a lattice, and $\bar{P}_{+}$ is the average power for $x>0$, and $\bar{P}_{-}$ is the average power for $x<0$. Therefore, when $\eta <0.5$, the surface wave mainly travels to $x<0$ direction, and $\eta >0.5$, surface wave mainly propagates to right. In fact, the power oscillates periodically, and the oscillation period is the lattice constant *a*, which is an amazing result because power should be a constant. This can be understood because the energy of the surface field in the air and the surface field in the PC slab is mutually converted, therefore, the total energy is still conserved. In a two-dimensional PC, the power oscillates with a period of *a* and the peak value of the power in the air corresponds to the valley value of the power in the PC. Finally, we obtain the spin separation is $\eta =0.003$ for the parameters used in Fig. 3c.

**Figure 5:**
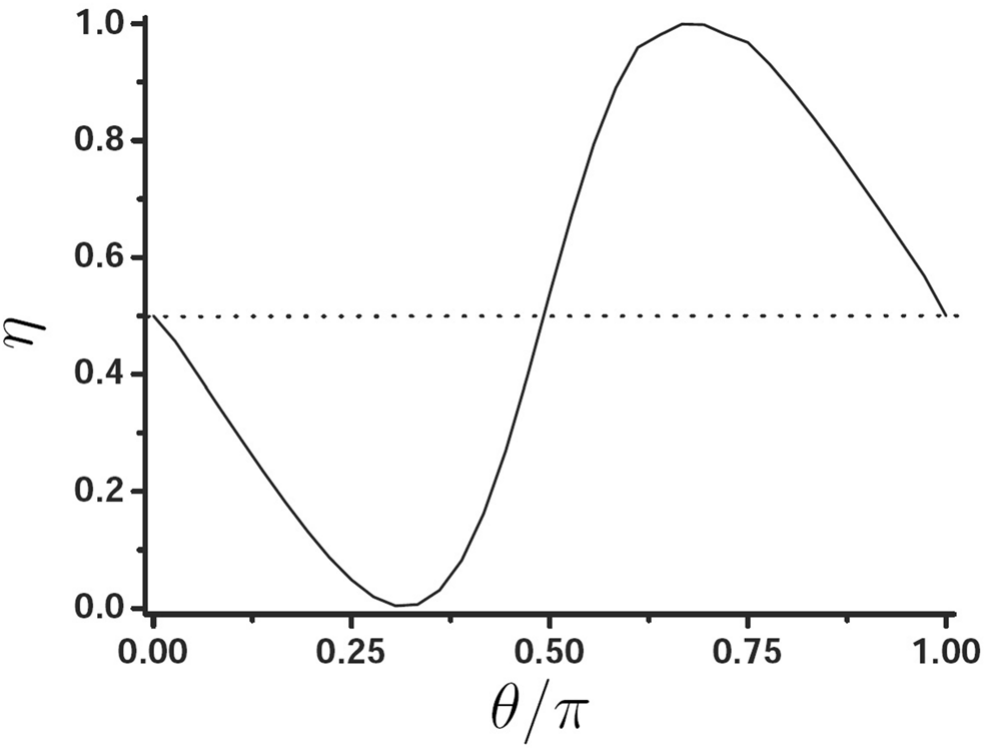
Spin separation as a function of polarization angle $\theta$.

Figure 5 presents the spin separation as a function of polarization angle $\theta$. We find that when $\theta =58^{\circ }$ and $115^{\circ }$, the spin separation is the optimal, this denotes the optimal PSHE is an elliptically polarized light, not a circularly polarized light. We also calculate the variation of spin separation as a function of $\theta$ for $N=20$, we finally find the optimal spin separation is the same with the case of $N=6$, this indicates that the number of layer has little effect on the PSHE of surface wave.

**Figure 6:**
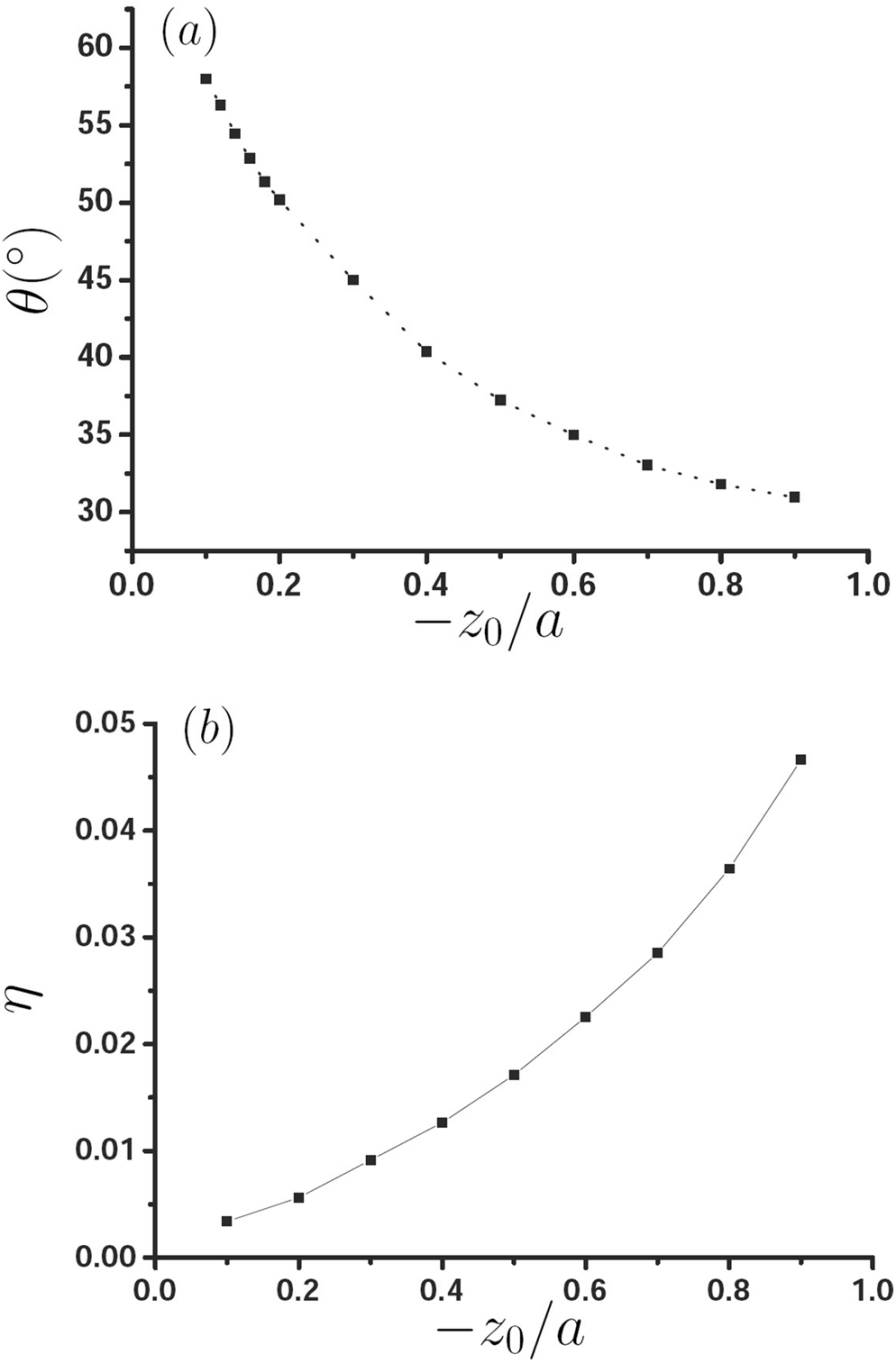
(**a**) Variation of the optimal $\theta$ with $-z_{0}$, (**b**) the optimal spin separation as a function of $-z_{0}$.

Now, we discuss the influence of the magnetic dipole position $z_0$ on PSHE. Figure 6 shows that in the case of the optimal PSHE, $\theta$ and the corresponding $\eta$ vary with $-z_0$. It can be seen that with the increase of the distance between the source with the interface, the optimal $\theta$ is decreased and the corresponding the spin separation is increased. That is to say, the smaller the distance between $\vec{m}$ and interface is, the better the PSHE is, this because with the decrease of the distance between the dipole and the interface, the corresponding interface field strength becomes stronger.

## Summary

The mechanism of asymmetric scattering of PSHE in PC slab is investigated, and we find that it is caused by the interference of the component surface waves excited by the different components of the incident light. Different from the SPP, the average spin with the orbit is locked for the surface field in PC slab. In addition, we found that the spin separation degree depends strongly on the polarization and position of the magnetic dipole, but the layer of the slab has little effect on the PSHE.

## Supplementary Information

Supplementary Information.
