# Supplementary material for: Unidirectional propagation of the Bloch surface wave excited by the spinning magnetic dipole in two-dimensional photonic crystal slab
Source: Sci Rep. 2021 Sep 16;11:18452. doi: 10.1038/s41598-021-98056-y (PMC8445978; doi:10.1038/s41598-021-98056-y)
Supplement: Supplementary file 1 — Supplementary Information. [file 41598_2021_98056_MOESM1_ESM.pdf]

Supplementary Materials for
Unidirectional propagation of the Bloch surface wave excited by the spinning magnetic dipole in two-dimensional photonic crystal slab

Li-Ming Zhao* and Yun-Song Zhou†

Department of Physics, Capital Normal University, Beijing 100086 China

TRANSFER MATRIX IN 2D PC

Now, we consider a 2D PC which consists of a square lattice (with lattice constant a) of dielectric cylinders (with radius r). The periodic plane layers are laid on the xz plane and cylinders are along the y-direction, and the periodic layers along z-direction may be finite, while along x-direction is infinite. For the case $E-polarized$ mode (the electric field are along y-direction), the Maxwell Equation can be written as

$$\frac{\partial^2 E}{\partial x^2} + \frac{\partial^2 E}{\partial z^2} + \epsilon(x,z)k_0^2 E = 0, \tag{A1}$$

as $\epsilon(x,z)$ is a periodic functions along x direction, so we can expand it in terms of a Fourier series as

$$\epsilon(x,z) = \sum_{G_x} \epsilon(G_x, z) e^{iG_x x}, \tag{A2a}$$

where G_x denotes the reciprocal lattice vector; $\epsilon(G_x, z)$ is the Fourier expansion components of $\epsilon(x,z)$. Now, we divide the slab into many slices and in each slice, the distribution of dielectric constant can be regarded as unchanged, in $l-th$ slice, the electric field can be written as

$$E(x,z) = \sum_{G_x} b(G_x) e^{i(k_x+G_x)x} e^{i\beta(z-z_{l-1})}, \tag{A2b}$$

Substituting Eqs. (A2a) and (A2b) into Eq. (A1), we can obtain

$$\sum_{G_x} [\epsilon(G_x^{'} - G_x, z)k_0^2 - (k_x + G_x^{'})^2 \delta_{G_x, G_x^{'}}] b(G_x) = \beta^2 b(G_x). \tag{A3}$$

By solving the eigen equation Eq. (A3), the eigen value β and eigen function $b(G_x)$ can be obtained. Therefore, the formal solution in the $l-th$ slice can be rewritten as

$$E^{(l)} = \sum_j A_l^{(j)} \sum_{G_x} b_l(G_x) e^{i(k_x+G_x)x} e^{i\beta_j(z-z_{l-1})} + \sum_j B_l^{(j)} \sum_{G_x} b_l(G_x) e^{i(k_x+G_x)x} e^{-i\beta_j(z-z_{l-1})}. \tag{A4}$$

Now, we assume each slice is adjacent to zero-thickness air, and the formal solution for the air layer can be written as

$$E_{l+} = \sum_{G_x} [A_{l+} e^{i(k_x+G_x)x} e^{i\beta_g(z-z_l)} + B_{l+} e^{i(k_x+G_x)x} e^{-i\beta_g(z-z_l)}],$$

$$E_- = \sum_{G_x}[A_{l-}e^{i(k_x+G_x)x}e^{i\beta_g(z-z_{l-1})} + B_{l-}e^{i(k_x+G_x)x}e^{-i\beta_g(z-z_{l-1})}], \quad (A5)$$

By employing the matching technique, we can obtain that

$$\begin{pmatrix} A_{l+} \\ B_{l+} \end{pmatrix} = S_2 S_1^{-1} \begin{pmatrix} A_{l-} \\ B_{l-} \end{pmatrix}$$

where,

$$S_1 = \begin{bmatrix} b_l \frac{\beta_g+\beta}{2\beta_g} & b_l \frac{\beta_g-\beta}{2\beta_g} \\ b_l \frac{\beta_g-\beta}{2\beta_g} & b_l \frac{\beta_g+\beta}{2\beta_g} \end{bmatrix}$$

and

$$S_2 = \begin{bmatrix} b_l \frac{\beta_g+\beta}{2\beta_g} e^{i\beta d_l} & b_l \frac{\beta_g-\beta}{2\beta_g} e^{-i\beta d_l} \\ b_l \frac{\beta_g-\beta}{2\beta_g} e^{i\beta d_l} & b_l \frac{\beta_g+\beta}{2\beta_g} e^{-i\beta d_l}, \end{bmatrix}$$

here, we can derive a transfer matrix that establishes the connection of the field amplitudes of the exit wave of $l-th$ layer A_{l+}, B_{l+} with the incoming wave of the $l-th$ layer A_{l-}, B_{l-}. It is noting that the A_{l-} (B_{l-}) is also the $A_{(l-1)+}$ ($B_{(l-1)+}$) and A_{l+} (B_{l+}) is $A_{(l+1)-}$ ($B_{(l+1)-}$). Another numerically more stable way is to use the scattering-matrix formulation. The details about the transformation technique from transfer matrix to scattering-matrix can be referred to [27,28]. Finally, relationship of A_N and B_1 with A_1 and B_N can be written as

$$\begin{pmatrix} A_N \\ B_1 \end{pmatrix} = S \begin{pmatrix} A_1 \\ B_N \end{pmatrix} \quad (A6)$$

REFLECTION IN 2D PC SLAB

Now, we investigate the reflectivity of light in a 2D PC slab which is located at region II and surrounded by dielectric medium on either side (referred to as regions I and III), as shown in Figure 1. We assume that a plane light wave is launched upon the surface of the PC slab from its left-side. The coordination of the interface between regions I and II is chosen to be $z = 0$. It is assumed that N represents the number of periodic plane layers in the PC slab.

When the incident plane wave with $E_{in} = E_0 e^{i[k_x x + k_z z]}$ illuminates the 2D PC slab, a set of real or complex Bloch modes may be excited. The EM field in region I is a superposition

of the incident plane wave and the reflected Bragg waves, whereas in region III, the EM field only contains the transmitted Bragg waves by considering the scattering condition. Therefore, the formal solution in Region I and III can be written as

$$E_1(x,z) = e^{i[k_x x + k_z z]} + \sum_n R_n e^{i[(k_x+nG_x)x + k_{nz} z}], \qquad (A7)$$

and

$$E_3(x,z) = \sum_n T_n e^{i[(k_x+nG_x)x + k_{nz}(z-z_N)]}. \qquad (A8)$$

By using Eq. (A6), we can obtain the reflection and transmission coefficients as

$$\begin{pmatrix} T \\ R \end{pmatrix} = S \begin{pmatrix} A_1 \\ 0 \end{pmatrix} \qquad (A9)$$

here $A_1 = (0, 0, ...1, ..., 0)$, $T = (..., T_{-1}, T_0, T_1, ...)$, $R = (..., R_{-1}, R_0, R_1, ...)$.

---

* zlm-phy@cnu.edu.cn

† 263zys@263.net
